# Regression of uremic tumoral calcinosis following the switch from cinacalcet to etelcalcetide in a hemodialysis patient without severe hyperphosphatemia

**DOI:** 10.1007/s13730-026-01181-8

**Published:** 2026-10-09

**Authors:** Takuya Hase, Shunsuke Goto, Hitomi Hara, Keisuke Oe, Tomoaki Fukui, Hideki Fujii

**Affiliations:** 1 Division of Nephrology, Kobe University Graduate School of Medicine, 7-5-2, Kusunoki-cho, Chuo-ku, Kobe, Hyogo 650-0017 Japan; 2 Department of Orthopaedic Surgery, Kobe University Graduate School of Medicine, Kobe, Japan

**Keywords:** Case report, Tumoral calcinosis, Hemodialysis, Etelcalcetide

## Abstract

Tumoral calcinosis (TC), a rare disease characterized by calcified mass formation in peri-articular soft tissue, manifests clinically as fractures, pain, and limited range of motion. In hemodialysis patients, severe hypercalcemia and hyperphosphatemia are major causes of the condition, and correcting these metabolic disorders reportedly induces its regression. Herein, we report the case of a 46-year-old man on maintenance hemodialysis who developed TC that regressed following etelcalcetide therapy despite the absence of severe hyperphosphatemia. The patient had started hemodialysis at age 32, and soft tissue calcifications were first noted two years later. These progressed to TC, which caused a left femoral shaft fracture 13 years after the initiation of dialysis. When the fracture occurred, TC was present in both thighs and the left elbow. Seven months after the fracture, he was referred to our department. Laboratory findings showed serum phosphate, corrected calcium, and intact parathyroid hormone levels of 4.9 mg/dL, 10.3 mg/dL, and 109 pg/mL, respectively. Cinacalcet was switched to intravenous etelcalcetide hydrochloride, and significant regression of the remaining TC was observed six months after the switch. Our findings suggest that etelcalcetide may be effective for TC in patients on hemodialysis, even in the absence of severe hypercalcemia or hyperphosphatemia. However, further reports are required to determine whether this observation is generalizable.

**Supplementary Information:**

The online version contains supplementary material available at https://doi.org/10.1007/s13730-026-01181-8.

## Introduction

Tumoral calcinosis (TC) is a rare disorder characterized by the deposition of calcified masses composed of calcium salts in the peri-articular soft tissues [1]. It typically involves large joints such as the elbows, hips, wrists, and shoulders, and may present as solitary or multiple lesions. The most common manifestations include restricted joint mobility and neurological impairment due to a local mass effect. Macroscopically, these lesions often exhibit a multilobulated configuration. They consist of calcium and hydroxyapatite deposits [2].

The etiology of TC is heterogeneous, encompassing hereditary disorders, prior tissue injury, primary hyperparathyroidism, vitamin D intoxication, and scleroderma [1]. Uremia is also a recognized cause, although the condition is rarely encountered in patients on hemodialysis. The precise pathogenesis remains incompletely understood; however, in dialysis patients, severe hyperphosphatemia and hypercalcemia are considered major determinants. Previous case reports have documented the regression of TC in dialysis patients following calcium- and/or phosphate-lowering interventions [1, 3, 4]. In contrast, therapeutic strategies in patients without marked hypercalcemia or hyperphosphatemia have seldom been described.

In this paper, we report a case of uremic TC that regressed with etelcalcetide despite only mild elevations in serum calcium and phosphate levels.

## Case report

A 46 year-old man was referred to our department for management of TC. He started maintenance hemodialysis at the age of 32 for end-stage renal disease of unknown etiology. Two years after hemodialysis initiation, soft tissue calcifications were noted, which subsequently increased in size and became multifocal.

Thirteen years after starting dialysis, he developed pain in the left thigh and was transferred to our emergency department. Radiographic examination revealed a left femoral shaft fracture possibly caused by TC, as well as calcified lesions in both thighs and the left elbow. He underwent proximal femoral replacement with resection of the TC.

**Fig. 1:**
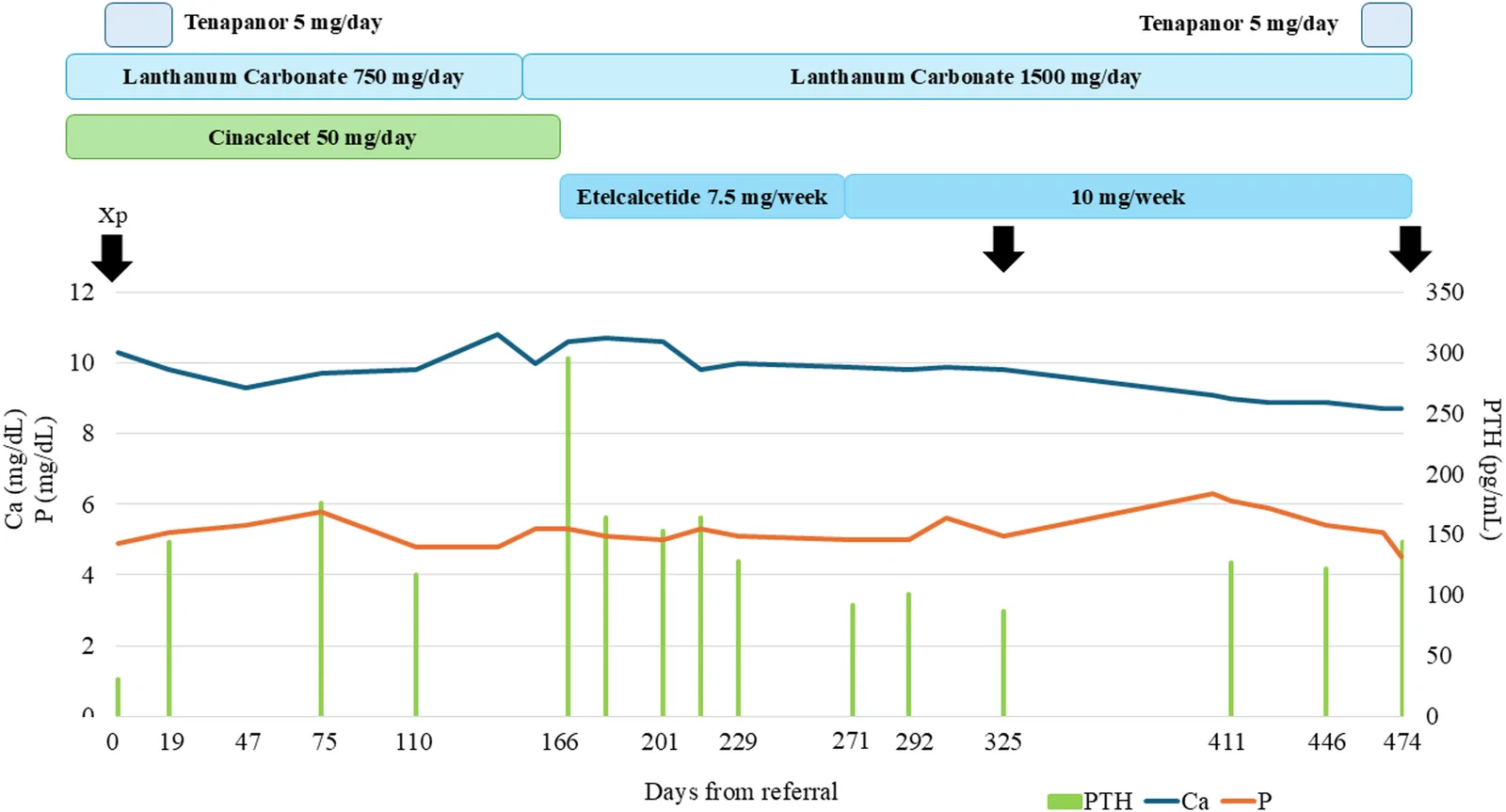
Time course of serum parathyroid hormone (PTH), calcium (Ca), and phosphate (P) levels during follow-up. The green bars represent serum intact PTH levels (pg/mL, left Y-axis), the blue line indicates serum calcium levels (mg/dL, right Y-axis), and the orange line shows serum phosphate levels (mg/dL, right Y-axis)

Four months later, he was readmitted with right femoral pain due to an impending fracture secondary to TC, for which intramedullary nailing was performed. Three months after the procedure, he was referred to our department, where laboratory exploration revealed serum phosphate, corrected calcium, intact parathyroid hormone (PTH), and serum alkaline phosphatase levels of 4.9 mg/dL, 10.3 mg/dL, 109 pg/mL, and 65 U/L, respectively. During the approximately 1.5 years preceding referral, serum phosphate, calcium, and intact PTH levels ranged from 4.0 to 6.5 mg/dL, 9.5 to 12.5 mg/dL, and 50 to 150 pg/mL, respectively. He had been receiving cinacalcet hydrochloride (50 mg/day) and lanthanum carbonate (750 mg/day) for the management of hyperparathyroidism and hyperphosphatemia. Tenapanor hydrochloride was initiated to achieve strict control of serum phosphate levels, although the patient’s serum phosphate levels were within the target range according to the Japanese guidelines (serum phosphate level of 3.5–<5.5 mg/dL, corrected serum calcium level of 8.4–<9.5 mg/dL, and intact PTH level of < 240 pg/mL). However, the medication was promptly discontinued because of intolerable diarrhea. Subsequently, the lanthanum carbonate dose was increased from 750 to 1500 mg, but his serum phosphate levels remained unchanged (Fig. 1).

Five months after referral to our department, it was recognized that blood samples had been collected several hours after oral cinacalcet administration, suggesting that his hypercalcemia and intact PTH levels might have been underestimated. Therefore, cinacalcet (50 mg/day) was switched to intravenous etelcalcetide hydrochloride (7.5 mg/week). Eight months after referral, the dose of etelcalcetide was increased to 10 mg/week.

**Fig. 2:**
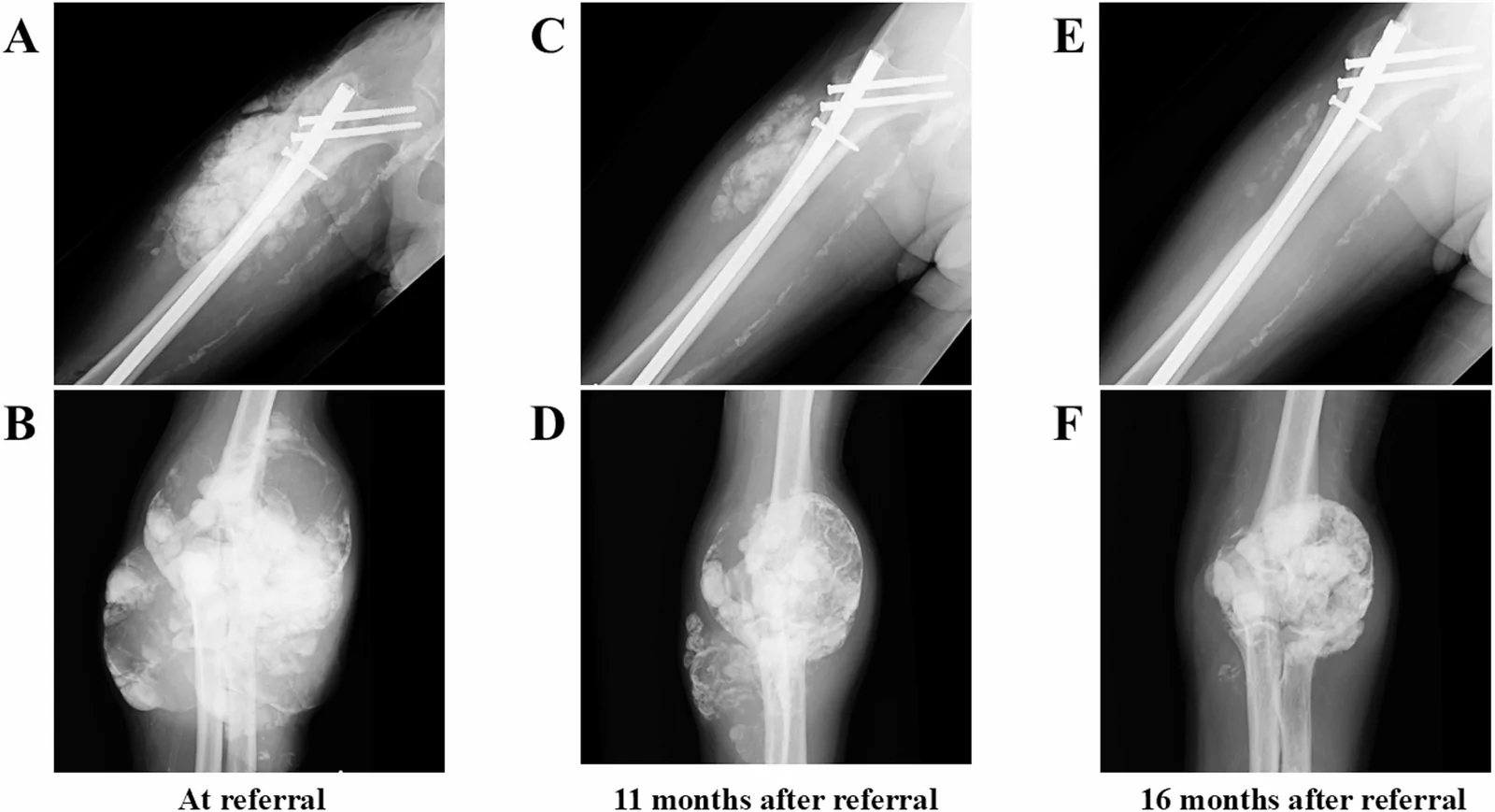
Serial radiographic changes in tumoral calcinosis in the right thigh and left elbow after referral. **A**, **B** At referral. **C**, **D** 11 months after referral. **E**, **F** 16 months after referral

Eleven months after referral, laboratory exploration revealed serum phosphate, corrected calcium, intact PTH, and alkaline phosphatase levels of 5.1 mg/dL, 9.8 mg/dL, 86.7 pg/mL, and 55 U/L, respectively. Radiographic examination revealed the regression of the calcified lesions in the right thigh and left elbow (Fig. 2). Tenapanor hydrochloride was reinitiated 15 months post-referral. Two weeks later, the serum phosphate, corrected calcium, and intact PTH levels were 4.5 mg/dL, 8.7 mg/dL, and 144.7 pg/mL, respectively, and the TC had regressed further.

## Discussion

TC is an uncommon complication in patients undergoing dialysis. Severe hypercalcemia and hyperphosphatemia are generally considered major contributors to its development, and conventional therapeutic strategies include phosphate-lowering therapy, parathyroidectomy, longer dialysis duration, reduction of dialysate calcium concentration, and discontinuation of active vitamin D [1]. Calcimimetics have been reported as a treatment option in several case reports [5–8]. In these reports, patients typically presented with severe hypercalcemia and/or hyperphosphatemia (Table S1). In our case, however, TC regressed with calcimimetic treatment despite only mild elevations in serum calcium and phosphate levels.

One possible explanation is that the severity of the patient’s hyperparathyroidism may have been underestimated. Laboratory testing in this case was performed *approximately 5–7* hours after oral administration of cinacalcet hydrochloride. Cinacalcet is known to induce nadirs of serum calcium and PTH levels at approximately 8–12 h and 2–4 h, respectively, after its administration [9]. Therefore, the laboratory measurements may have coincided with a transient post-dose nadir, leading to an underestimation of the serum calcium and PTH levels. Based on the data from previous clinical trials, intact PTH and serum calcium levels may have been underestimated by approximately 100 pg/mL and 0.2 mg/dL, respectively.

Switching from cinacalcet to etelcalcetide may have contributed to the regression of TC in our case. Etelcalcetide has been shown to provide a more sustained reduction in serum calcium and PTH levels in patients on hemodialysis [10]; however, as noted above, the effects of cinacalcet partially diminish after the serum levels of calcium and PTH reach their nadir [9]. Thus, the more sustained suppression of serum calcium achieved with etelcalcetide may have played a role in the regression of TC observed in our patient. Furthermore, although poor adherence to cinacalcet was not apparent in this case, switching to intravenously administered etelcalcetide during hemodialysis may have ensured more consistent and reliable drug administration.

In our case, vascular calcification remained unchanged despite TC regression (Fig. 2). In an animal study, cinacalcet was shown to suppress the progression of aortic calcification in 5/6 nephrectomized rats. [11]. However, a randomized controlled trial demonstrated that cinacalcet combined with low-dose vitamin D sterol did not induce the regression of coronary artery calcification in hemodialysis patients, although the progression of calcification was attenuated compared with flexible vitamin D therapy [12]. Similarly, a single-arm clinical study reported that aortic calcification continued to progress in patients treated with cinacalcet, albeit at a slower rate [13]. Taken together, these findings suggest that calcimimetics may be insufficient to achieve the regression of vascular calcification. The regression of TC observed in our case highlights the possibility of the mechanisms underlying soft tissue calcification and vascular calcification being distinct.

Although TC resulted in bilateral femoral fractures in our case, skeletal involvement is generally rare. In a case series of 16 patients with TC, only one patient exhibited mild iliac bone erosion associated with a pelvic lesion [1]. Nevertheless, a previous case report described intraosseous extension of TC leading to a femoral neck fracture [14]. These findings suggest that, although uncommon, TC can occasionally extend to bone and result in pathological fractures, a possibility that should be carefully considered in clinical practice. In our case, CT images showed that tumoral calcinosis was present at the fracture sites. Therefore, although the precise mechanism remains unclear, tumoral calcinosis may have contributed to the fractures, possibly through external pressure on the femur.

In conclusion, we present a case of uremic TC demonstrating regression after switching from cinacalcet to etelcalcetide in the absence of pronounced hypercalcemia or hyperphosphatemia. Since serum calcium levels may fluctuate in patients treated with cinacalcet, careful consideration of the timing of blood sampling is warranted. Furthermore, the use of long-acting calcimimetics may be beneficial for uremic TC, even in patients without severe hypercalcemia or hyperphosphatemia. However, further reports are required to determine whether this observation is generalizable.

## Supplementary Information

Below is the link to the electronic supplementary material.

Supplementary Material 1.
