# Supplementary material for: Regression of uremic tumoral calcinosis following the switch from cinacalcet to etelcalcetide in a hemodialysis patient without severe hyperphosphatemia
Source: CEN Case Rep. 2026 Oct 9;15(6):147. doi: 10.1007/s13730-026-01181-8 (PMC13653017; doi:10.1007/s13730-026-01181-8)
Supplement: Supplementary file 1 [file 13730_2026_1181_MOESM1_ESM.pdf]

Regression of uremic tumoral calcinosis by etelcalcetide in hemodialysis patients without severe hypercalcemia or hyperphosphatemia

Takuya Hase[1], Shunsuke Goto[1], Hitomi Hara[2], Keisuke Oe[2], Tomoaki Fukui[2], Hideki Fujii[1]

1. Division of Nephrology, Kobe University Graduate School of Medicine, Kobe, Japan

2. Department of Orthopaedic Surgery, Kobe University Graduate School of Medicine, Kobe, Japan.

Supplementary material

Supplementary Table S1.

Cases of tumoral calcinosis improved with calcimimetic therapy

No	Authors	Ca (mg/dL)	P (mg/dL)	PTH (pg/mL)	Treatment
1	Yamada S [1]	12.6	6.8	320	Cinacalcet
2	Moral Berrio E [2]	9.9	8.8	901	Cinacalcet
3	Sunder S [3]	10.1	8.8	1324	Cinacalcet
4	Hayat A [4]	10.4	8.4	384	Cinacalcet

Supplementary References

1. Yamada S, Tsuruya K, Kitazono T, Regression of tumoral calcinosis after the appropriate control of a deranged mineral and bone metabolism, in conjugation with cinacalcet hydrochloride treatment, in a chronic hemodialysis patient, Ther Apher Dial. 17 (3) (2013) 348-9, https://doi.org/10.1111/1744-9987.12035.
2. Moral Berrio E, Cox Conforme RA, Elías R, De La Flor JC, Rodríguez Tudero C, Sánchez de la Nieta-García MD, Zamora González-Mariño R, Vozmediano Poyatos C, An Unusual Case of Uremic Tumoral Calcinosis with Atypical Manifestation in a Patient on Peritoneal Dialysis: Case Report and Review of the Literature, Case Report and Review of the Literature. Med Sci (Basel). 13 (1) (2025) 11, https://doi.org/10.3390/medsci13010011.
3. Sunder S, Verma H, Venkataramanan K, Cervical tumoral calcinosis with secondary hyperparathyroidism in a chronic hemodialysis patient, Hemodial Int. 17 (3) (2013) 458-62, https://doi.org/10.1111/j.1542-4758.2012.00760.x
4. Hayat A. Complete Resolution of Tumoral Calcinosis in a Patient on Peritoneal Dialysis with Cinacalcet, Perit Dial Int. 39 (3) (2019) 292-293, https://doi.org/10.3747/pdi.2018.00212.
